# Effects of Oats (*Avena sativa* L.) on Inflammation: A Systematic Review and Meta-Analysis of Randomized Controlled Trials

**DOI:** 10.3389/fnut.2021.722866

**Published:** 2021-08-27

**Authors:** Sun Jo Kim, Cheol Woon Jung, Nguyen Hoang Anh, Suk Won Kim, Seongoh Park, Sung Won Kwon, Seul Ji Lee

**Affiliations:** ^1^College of Pharmacy, Seoul National University, Seoul, South Korea; ^2^Department of Statistics, Sungshin Women's University, Seoul, South Korea; ^3^Plant Genomics and Breeding Institute, Seoul National University, Seoul, South Korea

**Keywords:** *Avena sativa* L., inflammation, C-reactive protein, interleukin, dietary intervention

## Abstract

**Background:** Oat and its compounds have been found to have anti-inflammatory effects. Through this systematic review and meta-analysis, we aimed to determine an evidence-based link between oat consumption and inflammatory markers.

**Methods:** The Preferred Reporting Items for Systematic Reviews and Meta-Analyses (PRISMA) guidelines were followed. By the end of April 2021, we included randomized controlled trials (RCTs) that investigated the anti-inflammatory effect of oat and oat-related products through screening PubMed, Embase, Web of Science, ClinicalTrial.gov, and CENTRAL. Meta-analysis was conducted with a random-effect model on the standardized mean difference (SMD) of the change scores of inflammatory markers, including C-reactive protein (CRP), tumor necrosis factor-α (TNF-α), interleukin-6 (IL-6), and interleukin-8 (IL-8). Subgroup analyses were conducted to stratify confounding variables. The risk of bias was evaluated using the Cochrane risk of bias tool and Grading of Recommendations, Assessment, Development and Evaluation (GRADE) was applied to report the quality of evidence. This study was registered in the International Prospective Register of Systematic Reviews (PROSPERO; CRD42021245844).

**Results:** Systematic screening of five databases yielded 4,119 studies, of which 23 RCTs were finally selected. For the four systemic inflammatory markers analyzed, no significant alterations were found after oat consumption. However, oat intake was found to significantly decrease CRP levels in subjects with one or more health complications (SMD: −0.18; 95% CI: −0.36, 0.00; *P* = 0.05; *I*^2^ = 10%). Furthermore, IL-6 levels were significantly decreased in subjects with dyslipidemia (SMD = −0.34; 95% CI: −0.59, −0.10; *P* = 0.006; *I*^2^ = 0%). These beneficial effects might be attributed to the effects of avenanthramide and β-glucan.

**Conclusions:** Overall evidence supporting the alleviation of inflammatory response by oat intake was poor, calling for future studies including a larger sample size to confirm the findings.

## Introduction

Inflammation plays a pivotal role in the body's immune response to infection. Moreover, it maintains physiological homeostasis under a variety of abnormal conditions (1). However, excessive inflammation can cause various acute and chronic diseases, including atherosclerosis (2), autoimmune diseases (3), cancer (4), and depression (5). High-calorie diets, diets high in saturated fatty acids, and overeating increase the likelihood of abnormal inflammatory reactions (6, 7). The effects of diet control on inflammation are gaining research attention because diet constitutes a modifiable risk factor for inflammatory disorders. Thus, several studies have investigated the correlation between dietary habits and inflammation (8, 9).

Oats (*Avena sativa* L.) contain specific components, including avenanthramide, avenacoside, avenasterol, and β-glucan as major fiber (10, 11). Oats are widely consumed in the form of porridge and dietary supplements. Although several processing methods yield various commercial oat products, most products constitute whole grains (WG), because the processing methods for oat mostly preserve the germ and bran (12). Sufficient intake of WG is considered one of the cornerstones of a healthy diet owing to its numerous beneficial effects (13). For instance, WG supports maintaining physiological homeostasis by modulating inflammatory reactions (12, 14). Hence, oat and its components have been investigated and recognized as beneficial anti-inflammatory agents (15, 16). However, some studies have reported that oats actually have no anti-inflammatory effects (17, 18). Although there have been several meta-analyses on the anti-inflammatory properties of overall WG consumption (14, 19, 20), there is a lack of robust evidence in the absence of meta-analyses on the effects of oats and oat products on inflammation.

Therefore, we aimed to provide clinical evidence for the effects of oats on the modulation of inflammation by systematically inspecting randomized controlled trials (RCTs). To provide comprehensive information about oat effects, we included all data, regardless of their basal status, and all inflammatory markers or measures. Unprocessed oat, processed oat products, and oat-specific compounds were considered as appropriate intervention, whereas placebo diet, other ingredients, and a marginal amount of oat intake were considered as control.

## Materials and Methods

### Literature Search

This study followed the Preferred Reporting Items for Systematic Reviews and Meta-Analyses (PRISMA) guidelines (21) (Supplementary Table 1) and was registered in the International Prospective Register of Systematic Reviews (PROSPERO) (registration no. CRD42021245844). Detailed review protocol can be accessed in PROSPERO. All procedures were independently performed by at least two reviewers and the discrepancies were managed by a third-party reviewer. English literature was collected from five databases: PubMed, Embase, Web of Science, ClinicalTrial.gov, and CENTRAL to retrieve eligible studies.

An initial search date was March 30, 2021 and search results were regularly checked until April 30, 2021. The details of the search terms are listed in Table 1. Those who consumed a substantially small amount of oats and those who did not consume oats at all served as the controls. There was no limitation on the participants, allowing broad coverage of the discovery of inflammatory markers.

**Table 1:** Queries for the literature search.

**Group**	**Query**
Oat	*Avena sativa* OR *Avena sativa* L. OR Oat OR Avenanthramide OR Avenacin OR Avenoleic acid OR Avenasterol OR Avenacoside OR Desglucoavenacoside OR Quaker OR Porridge OR Avena OR Cultivated oat OR Cultivated oats OR Oat, Cultivated OR oats OR Oats, Cultivated OR Oat extract OR Oat milk OR Oatmeal OR Oat bran OR oat fiber OR AVA
Inflammatory markers	Inflammation OR Inflammatory biomarker OR Interleukin-10 OR IL-10 OR Interleukin-8 OR IL-8 OR Interleukin-6 OR IL-6 OR Interleukin-1β OR Interleukin-1 beta OR IL-1β OR IL-1β OR Interleukins OR Interleukin OR Inflammation mediator OR Tumor necrosis factor OR TNF OR C-reactive protein OR CRP OR High-sensitivity C-reactive protein OR hs-CRP OR Transforming growth factor-B OR Transforming growth factor beta OR TGF-B OR TGF-β OR Cytokines OR Cytokine OR Acute phase reactant OR Matrix metalloproteinase OR MMP OR E-selectin OR P-selectin OR Intercellular adhesion molecule-1 OR ICAM-1 OR Monocyte chemotactic protein 1 OR MCP-1 OR Neurogenic Inflammation OR Myokine OR Adipokine

### Inclusion and Exclusion Criteria

A study was considered eligible if it satisfied all the following items: (i) the study was designed as a RCT; (ii) oats, oat-related products, or oat-specific compounds were consumed orally in the treatment group; (iii) oats, oat-related products, or oat-specific compounds were absent or insignificantly consumed in the control group; and (iv) any inflammatory markers or measures thereof were evaluated. The exclusion criteria were as follows: (i) inappropriate intervention for the treatment group (oats were mixed with other ingredients not related to oats) and/or inappropriate intervention for the control group (diet contained a significant amount of oats); (ii) outcomes unrelated to inflammatory outcomes; (iii) not a RCT; (iv) duplicated or a part of a more extensive research included beforehand; and (v) irrelevant publication type such as a review, conference, abstract, or any other secondary scientific reports.

### Data Extraction

Study characteristics, including the first author's name, country, year of publication, design of RCT (crossover or parallel), health status and age range of participants, details of treatment and control intervention (sample size, formula, and dose), and treatment duration, were extracted from the selected RCTs. If a study did not specify the type of processed oats, it was automatically deemed as whole oats. In terms of outcome, any inflammation-related markers or measurements were extracted along the direction of alteration. If available, values of data distribution, such as mean, standard deviation (SD), standard error (SE), and 95% confidence interval (CI), were obtained.

### Meta-Analysis

Review Manager 5.4 (Nordic Cochrane Center, The Cochrane Collaboration) was used for the overall meta-analysis. The meta-analysis was conducted on commonly reported inflammatory markers, including C-reactive protein (CRP), tumor necrosis factor-α (TNF-α), interleukin-6 (IL-6), and interleukin-8 (IL-8). Markers reported in fewer than three studies were not examined due to the lack of statistical power. The overall effect sizes were calculated by synthesizing the difference in change scores. Accordingly, studies that provided either change scores within each group or both baseline and post-treatment levels were considered eligible. For crossover RCTs, the difference of two post-treatment values was used to infer intergroup differences based on the assumption that a wash-out period eliminated all carry-over effects and resulted in an identical baseline status. If the SD was not explicitly presented in the study, it was calculated by transforming the values of either SE or CI, considering that the data were normally distributed. Notably, the SE of the mean difference (MD) was determined depending on the presented data type (change score vs. baseline/post-treatment) and RCT design (parallel vs. crossover). To incorporate crossover RCT into the meta-analysis, the correlation coefficient between change scores was imputed to approximate a paired analysis. Meanwhile, the SD of each change score was calculated by imputing a correlation between baseline and post-treatment in the case of parallel RCT. Since no study presented necessary correlation information, all the unknown correlation coefficients were set to 0.5. This was in accordance with previous studies (22, 23). Because of an observable heterogeneity in the magnitude of values, the effect sizes were expressed as the standardized mean difference (SMD). The equations for the SE of MD, SMD, and SE of SMD calculation are presented in Table 2. The random-effects method with an inverse-variance approach was applied. Heterogeneity between studies was estimated using Cochran's Q test and *I*^2^. Robust statistics were examined by sensitivity analyses using the leave-one-out method and by imputing correlation coefficients (ρ) of 0.2 and 0.8. The most representative oat and control groups were chosen over others if multiple groups were presented. An intervention period of at least 2 weeks was considered eligible to observe treatment-induced effects. If the measurement of markers was performed at multiple time points, the most approximate period to those of the other studies was selected. Subgroup analyses were conducted to stratify confounding variables, including the type of measurement (CRP vs. hs-CRP), basal condition (healthy vs. unhealthy), type of oat product (whole vs. fiber-rich fraction), and type of control (placebo or no intervention vs. other materials, such as wheat). However, subgroup analyses of TNF-α and IL-8 were not conducted because the number of included RCTs was limited (*n* = 3 for both). Studies that provided only median over the mean or interquartile range over SD were excluded. A funnel plot was generated for each meta-analyzed marker to visualize the potential publication bias. Two-tailed *p*-values were estimated following Begg's rank correlation test and Egger's regression test to evaluate funnel plot asymmetry. A *p*-value <0.1 was considered an inherent risk for publication bias.

**Table 2:** Calculation of the standard error (SE) of the mean difference (MD), the standardized mean difference (SMD), and the SE of SMD based on the type of study design (parallel vs. crossover randomized controlled trials).

	**Parallel**	**Crossover**
*SE of MD* (change score^†^)	$\sqrt{\frac{\sigma_{d_{T}}^{2}}{N_{T}}+\frac{\sigma_{d_{C}}^{2}}{N_{C}}}$	–
*SE of MD* (pre-/post-)	$\sqrt{\frac{\sigma_{T_{1}}^{2}}{N_{T_{1}}}+\frac{\sigma_{T_{2}}^{2}}{N_{T_{2}}}-\frac{2\rho_{T_{1},T_{2}}\sigma_{T_{1}}\sigma_{T_{2}}}{\max\left(N_{T_{1}},N_{T_{2}}\right)}+\frac{\sigma_{C_{1}}^{2}}{N_{C_{1}}}+\frac{\sigma_{C_{2}}^{2}}{N_{C_{2}}}-\frac{2\rho_{C_{1},C_{2}}\sigma_{C_{1}}\sigma_{C_{2}}}{\max\left(N_{C_{1}},N_{C_{2}}\right)}}$	$\sqrt{\frac{\sigma_{T_{1}}^{2}}{N_{T_{1}}}+\frac{\sigma_{C_{1}}^{2}}{N_{C_{1}}}-\frac{2\rho_{T_{1},C_{1}}\sigma_{T_{1}}\sigma_{C_{1}}}{\max\left(N_{T_{1}},N_{C_{1}}\right)}}$
*SD*_*pooled*_	$\sqrt{\frac{\sigma_{d_{T}}^{2}+\sigma_{d_{C}}^{2}}{2}}$	$\sqrt{\frac{\sigma_{T_{1}}^{2}+\sigma_{C_{1}}^{2}}{2}}$
SMD	$\frac{MD}{SD_{pooled}}$	$\frac{MD}{SD_{pooled}}$
*SE of SMD*	$\frac{SE\;of\;MD}{SD_{pooled}}$	$\sqrt{2\left(\frac{1}{N}+\frac{SMD^{2}}{2N}\right)\left(1-\rho_{T_{1},\;C_{1}}\right)}$

*Change score:^†^ data presented as change score value*.

*Pre-/post-: data presented as pre-treatment (baseline) and post-treatment value*.

*T_1_: state of post-treatment in the oat-treated group*.

*T_2_: state of pre-treatment (baseline) in the oat-treated group*.

*d_T_: change (post—pre) in the oat-treated group*.

*C_1_: state of post-treatment in the control-treated group*.

*C_2_: state of pre-treatment (baseline) in the control-treated group*.

*d_C_: change (post—pre) in the control-treated group*.

*σ_A_: standard deviation of A state*.

*N_A_: sample size (population) of A state*.

*ρ_A, B_: correlation coefficient between A and B states*.

### Risk of Bias and Quality of Evidence Evaluation

The risk of bias in individual studies was evaluated using the second version of the Cochrane risk of bias tool for randomized trials (RoB2) (24). The tool had five bias domains, which contained at least three signaling questions that could address almost all the important aspects possibly influencing the results of a trial. In detail, the five bias domains evaluate bias that can arise from the randomization process, due to deviations from the intended intervention, missing outcome data, or occur in the measurement of the outcome and in the selection of the reported result. Each domain was regarded as either high risk, some concerns, low risk of bias. Finally, overall risk was determined based on evaluated domains of individual trials. The evaluation was independently conducted by two reviewers.

The quality of evidence on each meta-analyzed marker was evaluated based on the Grading of Recommendations, Assessment, Development and Evaluation (GRADE) (25). Since the study design of all the included studies was RCT, the quality estimate started from high quality and downgraded following the judgements to risk of bias, inconsistency, indirectness, imprecision, and publication bias.

## Results

### Search Results

**Figure 1:**
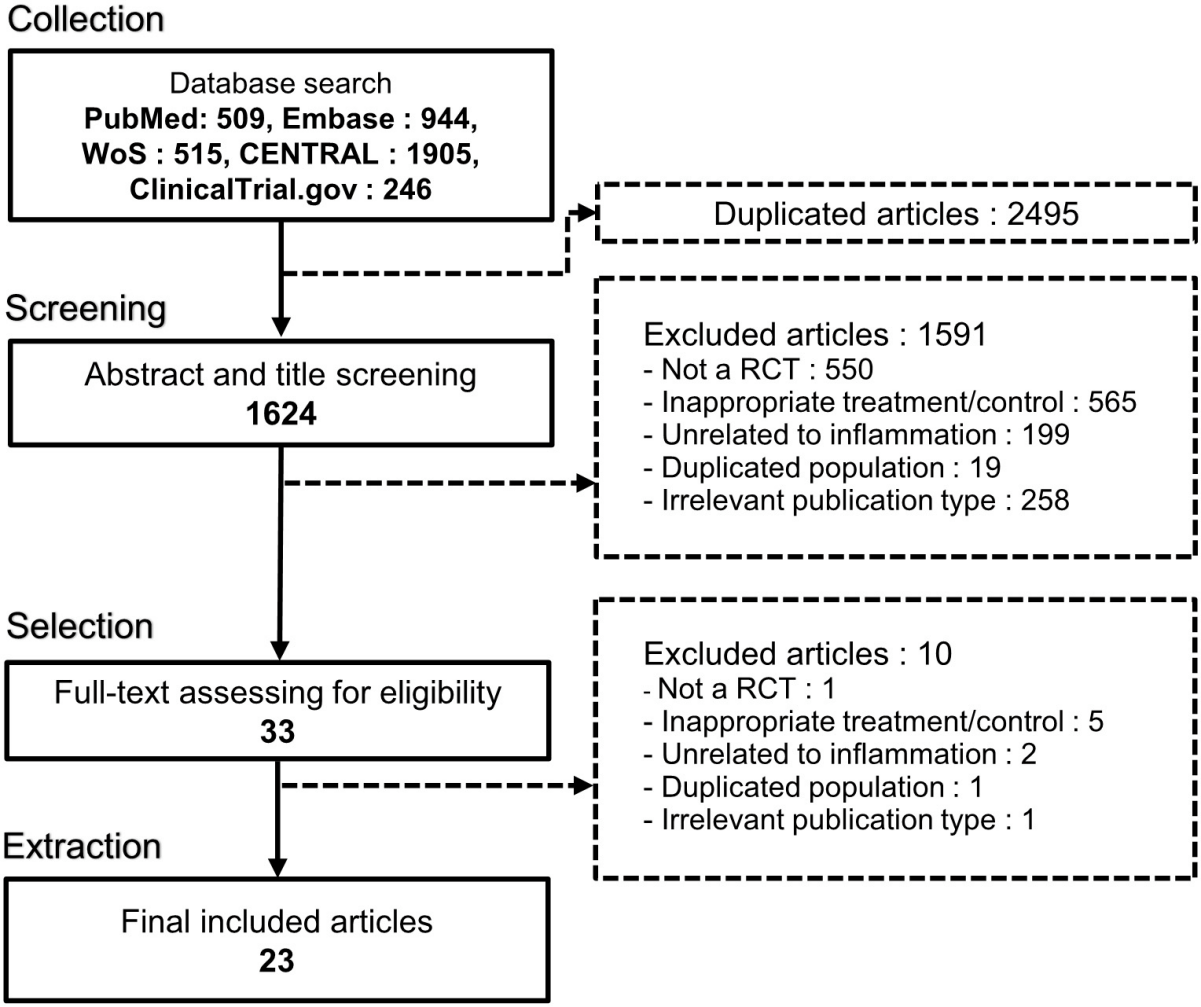
PRISMA flowchart of the study selection process.

Through systematic screening of the five databases, 4,119 studies were retrieved, of which 1,624 remained after duplication removal. Their titles and abstracts were then screened, and 1,591 studies were eliminated following the exclusion criteria. The full texts of the 33 remaining studies were examined to determine their eligibility, and 10 studies were excluded. Finally, 23 RCTs were selected for systematic review and meta-analysis (16–18, 26–45). The detailed workflow is shown in Figure 1.

### Study Characteristics and Summary of Outcomes

The studies included in this systematic review were published between 2008 and 2020. The sample size ranged from 16 to 362 participants. Among the included RCTs, 16 were conducted on parallel groups (16, 17, 26–28, 30–34, 37–39, 42, 43, 45), while the others were crossover designs. Five studies recruited only male or female subjects (16, 17, 27, 38, 39), while the remaining studies included both sexes. The studies mostly comprised healthy subjects, followed by patients with dyslipidemia and type 2 diabetes. In terms of treatment types, ten studies provided oats in the form of a fiber-rich fraction, including oat β-glucan and oat bran (17, 18, 26, 30–32, 37, 38, 40, 43). Nine studies provided products containing whole oats (28, 29, 33, 35, 36, 41, 42, 44, 45), while the others used avenanthramides (16, 27, 34) or oat protein (39). The duration of treatment was at least 2 weeks, except for the study by Sawicki et al. (41), wherein the short-term response of participants was investigated 1 day after the intake of oats.

**Figure 2:**
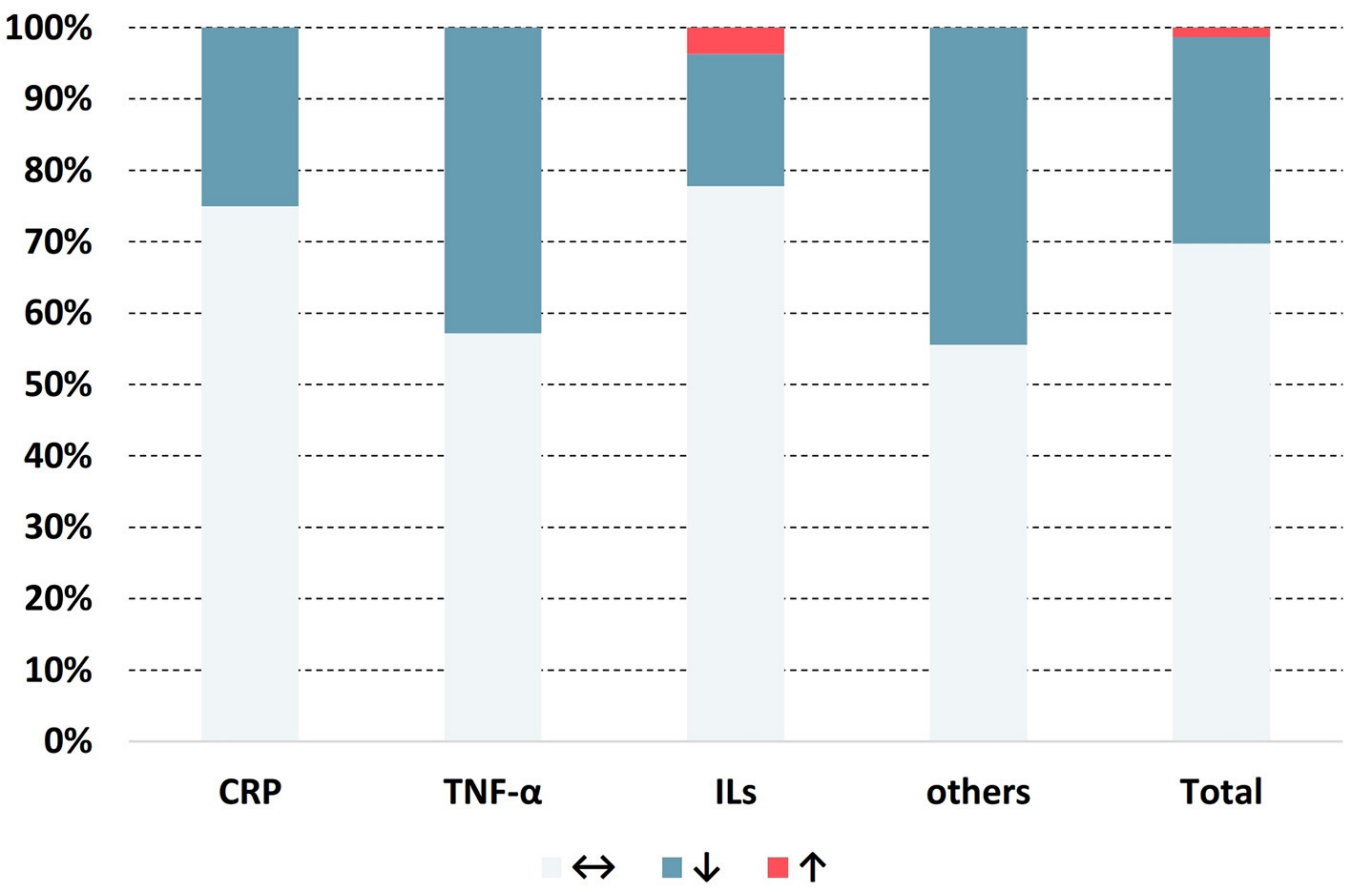
Distribution of the change in direction for anti-inflammatory markers. ↔: no significant change; ↓: significant decrease (*p* < 0.05); ↑: significant increase (*p* < 0.05).

A total of 76 results on inflammatory markers from 23 studies were obtained, among which 53 showed no significant change, 22 revealed reductions, and only 1 showed an increase (Figure 2). Detailed information on the extracted data is provided in Table 3.

**Table 3:** Study design, characteristics, and summary of outcomes.

**Author, country, year**	**Design of randomized controlled trial**	**Participants**	**Intervention, number (male/female), daily dose**	**Control, number (male/female), daily dose**	**Duration**	**Effect**	**Risk of bias**
McGeoch et al. (29) United Kingdom	Crossover	Type 2 diabetes, 40–75 years	Oat-based product, 27 (18/9), 60–100 g	Standard diet, 27 (18/9), NA	8 weeks	↔ CRP, IL-18, Adiponectin	Some concerns
Koenig et al. (27) United States	Parallel	Young female aged 18–30	Avenanthramides in oat cookies, 8 (0/8), 9.2 mg	Avenanthramides; oat flour cookies, 8 (0/8), 0.4 mg	8 weeks	↓ NRB, IL-6, TNF-α, CRP ↔ IL-1b	Low risk
Koenig et al. (16) United States	Parallel	Female aged 50–80	Avenanthramides in oat cookies, 8 (0/8), 9.2 mg	Avenanthramides; oat flour cookies, 8 (0/8), 0.4 mg	8 weeks	↓ IL-1B, NRB, CRP ↔ IL-6, TNF-α	Low risk
Zhang T et al. (34) United States	Parallel	Healthy subjects, 23.0 ± 1.2 years	Avenanthramides in oat cookies, 12 (NA), 20.6 mg	Avenanthramides; oat flour cookies, 12 (NA), 0 mg	8 weeks	↓ IL-1Ra, sVCAM-1, G-CSF, NRB ↔ IL-6, MCP-1, CK	High risk
Nieman et al. (17) United States	Parallel	Healthy male subjects	Oat β-glucan in beverage, 19 (19/0), 5.6 g/600 mL	Cornstarch in beverage, 17 (17/0), 600 mL	14 days	↔ IL-6, IL-10, IL-8, IL-1Ra	Some concerns
Tighe P et al. (33) United Kingdom	Parallel	Middle aged (40–65 y), healthy subjects	Oat cereals, 70 (36/34), 60–80 g	Whole-grain cereals, 73 (38/35), 90–120 g	12 weeks	↔ hs-CRP, IL-6	Some concerns
Fazilaty et al. (26) Iran	Parallel	Multiple trauma, ≥18 years	Oat β-glucan, 20 (18/2), 3 g	Maltodextrin, 20 (18/2), 3 g	21 days	↑ IL-12 ↔ hs-CRP	Some concerns
Ma et al. (28) China	Parallel	Type 2 diabetes, 50–60 years	Organic naked oat with whole germ porridge, 65 (27/38), 50 g	Diet group, 61 (28/33), balance nutritional composition without oat	30 days	↔ hs-CRP	Some concerns
			Organic naked oat with whole germ porridge, 65 (27/38), 100 g	Diet group, 61 (28/33), balanced nutritional composition without oat	30 days	↓ hs-CRP	
Ganda Mall et al. (31) Sweden	Parallel	Healthy subjects, ≥65 years	Oat β-glucan powder, 15 (9/6), 12 g	Maltodextrin powder, 17 (9/8), 12 g	6 weeks	↔ IFN-γ, IL-10, CRP, IL-1b, IL-2, IL-6, IL-8, TNF-α, IL-12p70	Some concerns
				Arabinoxylan powder, 17 (9/8), 12 g	6 weeks	↔ IFN-γ, IL-10, CRP, IL-1b, IL-2, IL-6, IL-8, TNF-α, IL-12p70	
Sirtori et al. (37) Italy	Parallel	Moderate hypercholesterolemia	Oat fiber containing 25-28% β-glucan with casein bar, 22 (NA), 10.5 g	Cellulose with casein bar, 25 (10/15), 10 g	4 weeks	↔ Adiponectin, sICAM-1, IL-6, hs-CRP	High risk
			Oat fiber containing 25-28% β-glucan with casein bar, 23 (NA), 10.5 g	Cellulose with casein bar, 25 (11/14), 10 g	4 weeks	↔ Adiponectin, sICAM-1, IL-6, hs-CRP	
Connolly et al. (35) United Kingdom	Crossover	Healthy subjects, 19–60 years	Whole grain oat granola, 30 (11/19), 45 g	Non-whole grain cereal, 30 (11/19), 45 g	6 weeks	↔ CRP, TNF-α, IL-6, Calprotectin, Ig A	Low risk
Xia et al. (39) China	Parallel	Healthy male, 19.7 ± 1.1 years	Oat protein beverage, 8 (8/0), 25 g	Maltodextrin beverage, 8 (8/0), 25 g	19 days	↓ CRP, IL-6	Low risk
Thompson et al. (38) United States	Parallel	Healthy male, 18–30 years	β-Glucan (oat bran) powder, 9 (9/0), 3 g	Muffin mix powder, 11 (11/0), 3 g	4 weeks	↓ 24 h soreness score	High risk
Theuwissen et al. (18) Netherlands	Crossover	Mild hypercholesterolemia, 52 ± 11 years	Oat β-glucan in muesli, 30 (NA), 4.8 g	Wheat fiber in muesli, 30 (NA), 4.8 g	4 weeks	↔ TNF-α, IL-6, IL-8, hs-CRP	High risk
Pavadhgul P et al. (36) Thailand	Crossover	Hypercholesterolemia, 30–60 years	Oat porridge, 24 (NA), 70 g	Rice porridge, 24 (NA), 70 g	4 weeks	↓ hs-CRP, IL-6, IL-8, TNF-α, MCP-1	High risk
Zhang et al. (44) United Kingdom	Crossover	Type 2 diabetes, 40–75 years	Oat-enriched diet (commercial product), 22 (NA), 131 g	Non-oat product (daily meal), 22 (NA), NA	8 weeks	↔ P-selectin, CRP, IL-18	High risk
Sawicki CM et al. (41) United States	Crossover	Overweight or mildly obese, metabolically at-risk, 40–70 years	Whole oat flour muffins, 13 (8/5), 48 g	Refined wheat flour muffins, 13 (8/5), 48 g	1 day	↔ hs-CRP, IL-6, IL-8, TNF-α	Some concerns
Biörklund et al. (40) Sweden	Parallel	Healthy, but with mildly elevated serum cholesterol levels, 35–72 years	Oat β-glucan soup, 22 (NA), 4 g per 400 g soup	Placebo soup, 21 (NA), 0 g per 400 g soup	5 weeks	↔ hs-CRP	Some concerns
Sturtzel et al. (42) Austria	Parallel	Frail patients (57–98 years), who are geriatric hospital residents with multiple chronic diseases and require assistance for their daily life activities	Oat flakes (oat-bran product) blended into common daily meals, 15 (NA), 5.2 g	Ward's habitual diet, 15 (NA), NA	12 weeks	↓ CRP	Some concerns
Queenan et al. (32) United States	Parallel	Hypercholesterolemia, 22–65 years	Oat β-glucan powder in beverage, 35 (22/13), 6 g	Dextrose powder in beverage, 40 (28/12), 6 g	6 weeks	↔ CRP	Some concerns
Cugnet-Anceau et al. (30) France	Parallel	Type 2 diabetes, 30–75 years	Soup containing oat β-glucan, 29 (NA), 3.5 g in 400 g	Soup without oat β-glucan, 24 (NA), 0 g in 400 g	11 weeks	↔ CRP	Some concerns
Maki et al. (45) United States	Parallel	Overweight and obese adults	Whole-grain oat cereal, 77 (NA), 40 g (providing 3 g β-glucan)	Low-fiber breakfast/snack foods, 67 (NA), ~500 kcal	12 weeks	↔ hs-CRP	Some concerns
Wolever et al. (43) Canada	Parallel	Healthy subjects, 20–65 years	High-molecular-weight oat β-glucan-containing cereals, 81 (43/43), 3 g	Wheat bran-containing cereals, 87 (36/51), NA	4 weeks	↔ CRP	Some concerns
			Medium-molecular-weight oat β-glucan-containing cereals, 67 (33/34), 4 g		4 weeks	↔ CRP	
			Medium-molecular-weight oat β-glucan-containing cereals, 64 (27/37), 3 g		4 weeks	↔ CRP	
			Low-molecular-weight oat β-glucan-containing cereals, 63 (22/41), 4 g		4 weeks	↔ CRP	

*NA, not available; ↔, no significant change; ↓, significant decrease (p <0.05); ↑, significant increase (p < 0.05)*.

### Effect of Oats on Interleukins and TNF-α

**Figure 3:**
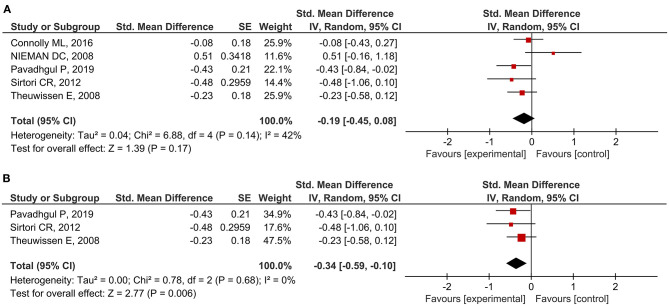
Meta-analysis results of IL-6: **(A)** all studies, **(B)** unhealthy subjects in the subgroup based on the basal condition.

A total of 27 outcomes for ILs were acquired from 15 RCTs (16–18, 26, 27, 29, 31, 33–37, 39, 41, 44). IL-6 was measured in 12 RCTs (16–18, 27, 31, 33–37, 39, 44), three of which reported a significant reduction (27, 36, 39), while the others did not observe any difference. Meta-analysis for IL-6 was conducted using the eligible data from five studies (17, 18, 35–37) (Table 4). The results showed that oats had no significant effect on circulating IL-6 levels (SMD = −0.19; 95% CI: −0.45, 0.08; *P* = 0.17; *I*^2^ = 42%; *N* = 167) (Figure 3A). In addition, introducing either 0.2 or 0.8 as a correlation coefficient did not change the outcome (Supplementary Tables 2, 3). However, we found a significant reduction when the subgroup was stratified into unhealthy subjects as a basal condition (SMD = −0.34; 95% CI: −0.59, −0.10; *P* = 0.006; *I*^2^ = 0%) (Figure 3B). This was consistent even when ρ = 0.2 and 0.8 were plugged-in for the unknown correlation parameter. All unhealthy subjects were patients with hypercholesterolemia. Notably, exclusion of study data from Nieman et al. (17) significantly improved the overall heterogeneity among the studies (42% to 0%) and significantly reduced SMD (SMD = −0.26; 95% CI: −0.46, −0.06; *P* = 0.01; *I*^2^ = 0%). In the case of IL-8, one out of five RCTs reported a significant reduction (36), and three of them provided eligible data for the meta-analysis (17, 18, 36). Oat intake was not correlated with IL-8 (SMD = −0.22; 95% CI: −0.71, 0.28; *I*^2^ = 70%; *N* = 90) (Supplementary Figure 1A). Similar to IL-6, the outcome did not change upon imputation of ρ = 0.2 and 0.8. Other markers, such as IL-β, IL-2, IL-10, IL-12, and IL-18, were mostly unchanged and could not be meta-analyzed due to the lack of eligible data.

**Table 4:** Summary of the results of this meta-analysis on C-reactive protein (CRP), interleukin-6 (IL-6), interleukin-8 (IL-8), and tumor necrosis factor alpha (TNF-α).

**Study group**	**Studies**	**Effect estimate**		**Heterogeneity**				**Grade**
		**Standard mean difference**	***p*-value**	***I*^**2**^ (%)**	**Q statistic**	**p-within**	**p-between**	
		**(95% CI)**			**group**	**group**	
**CRP**								
Overall	9	−0.12 [−0.28, 0.04]	0.15	11	9.04	0.34		Moderate*^a^*
Type of measurement		0.14	
CRP	2	0.14 [−0.26, 0.55]	0.49	23	1.29	0.26		
hs-CRP	7	−0.19 [−0.36, −0.02]	0.03	0	5.15	0.52		
Basal condition		0.16	
Healthy	2	0.07 [−0.23, 0.38]	0.65	0	0.32	0.57		
Unhealthy	7	−0.18 [−0.36, 0.00]	0.05	10	6.68	0.35		
Type of oat product		0.93	
Whole	4	−0.12 [−0.41, 0.17]	0.43	45	5.47	0.14		
Fiber-rich fraction	5	−0.10 [−0.32, 0.12]	0.37	0	3.49	0.48		
Type of control		0.15	
Placebo/no intervention	6	−0.11 [−0.38, 0.16]	0.42	35	7.68	0.17		
Other materials, such as wheat	3	−0.10 [−0.31, 0.11]	0.36	0	1.28	0.53		
**IL-6**	
Overall	5	−0.19 [−0.45, 0.08]	0.17	42	6.88	0.14		Low*^a^^,^^b^*
Basal condition		0.12	
Healthy	2	0.14 [−0.42, 0.70]	0.62	57	2.33	0.13		
Unhealthy	3	−0.34 [-0.59, −0.10]	0.006	0	0.78	0.68		
Type of oat product		0.67	
Whole	2	−0.24 [−0.58, 0.10]	0.17	38	1.60	0.21		
Fiber-rich fraction	3	−0.11 [−0.59, 0.38]	0.66	61	5.16	0.08		
Type of control		0.65	
Placebo/no intervention	2	0.00 [−0.97, 0.97]	1.00	79	4.80	0.03		
Other materials, such as wheat	3	−0.23 [−0.44, −0.02]	0.04	0	1.60	0.45		
**IL-8**	3	−0.22 [−0.71, 0.28]	0.40	70	6.65	0.04		Very low*^a^^,^^b^^,^^c^*
**TNF-α**	3	−0.14 [−0.41, 0.14]	0.33	39	3.29	0.19		Low*^a^^,^^b^*

*Downgraded for*

a *Risk of bias*,

b *Imprecision, and*

c *inconsistency*.

Although approximately half of the included RCTs (three out of seven) for TNF-α reported a significant reduction in TNF-α in oat-treated groups (16, 27, 36), the meta-analysis of three eligible data revealed that oats had no significant effect (SMD = −0.14; 95% CI: −0.41, 0.14; *I*^2^ = 39%; *N* = 84) (18, 35, 36). The result is presented in Supplementary Figure 1B. Sensitivity analysis did not reverse this outcome.

### Effect of Oats on CRP

**Figure 4:**
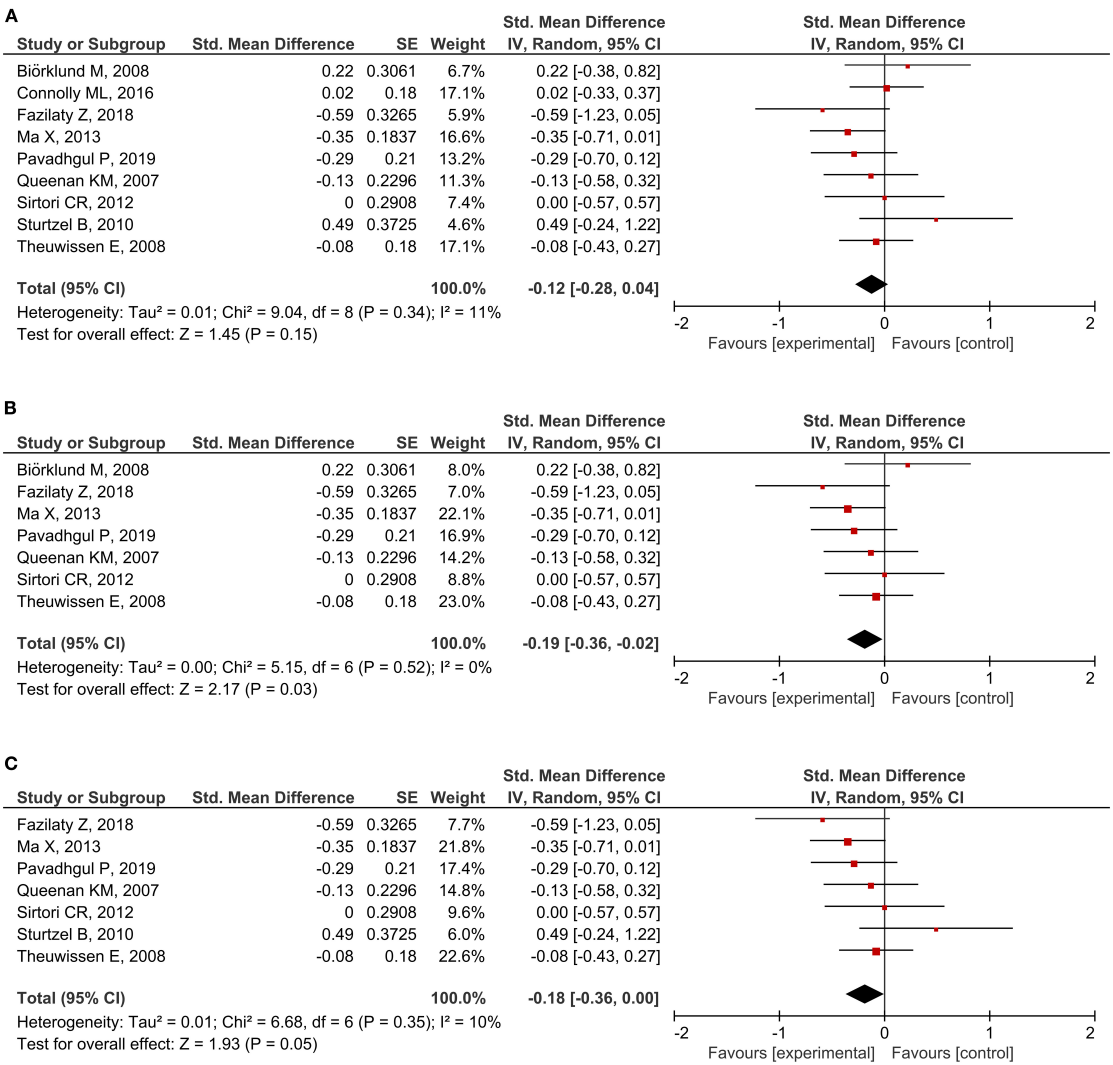
Meta-analysis results of CRP: **(A)** all studies, **(B)** hs-CRP in the subgroup based on the type of measurement, **(C)** unhealthy subjects in the subgroup based on the basal condition.

A total of 20 RCTs assessed CRP concentration, providing 24 CRP measurement results. Six results reported a significant reduction following oat intake (16, 27, 28, 36, 39, 42), whereas 18 showed insignificant change (18, 26, 28–33, 35, 37, 40, 41, 43–45). A meta-analysis was conducted based on eligible data from nine RCTs (18, 26, 28, 32, 35–37, 40, 42). Overall, the level of CRP was unchanged by oat intake (SMD = −0.12; 95% CI: −0.28, 0.04; *I*^2^ = 11%; *N* = 441) (Figure 4A). However, a significant reduction was observed when the measurement method was stratified to hs-CRP (SMD = −0.19; 95% CI: −0.36, −0.02; *P* = 0.03; *I*^2^ = 0%) (Figure 4B). Moreover, a subgroup analysis involving unhealthy subjects showed that CRP was generally decreased after intake of oats (SMD: −0.18; 95% CI: −0.36, 0.00; *P* = 0.05; *I*^2^ = 10%) (Figure 4C). There were no significant changes after the leave-one-out analysis and after the imputation of either 0.2 or 0.8 correlation coefficient.

### Effect of Oats on Other Inflammatory Markers

In addition to ILs, TNF-α, and CRP, several inflammatory markers were measured to examine the effects of oat intake on inflammation. However, we could not perform a meta-analysis on the other markers because of insufficient eligible data. Neutrophil respiratory burst (NRB), granulocyte colony-stimulating factor (G-CSF), and soreness score were consistently reduced after oat intake (16, 27, 34, 38). In contrast, there were no significant alterations in creatine kinase, interferon-gamma (IFN-γ), soluble intercellular adhesion molecule-1 (sICAM-1), immunoglobulin A (IgA), calprotectin, and P-selectin (31, 34, 35, 44). Contradicting outcomes were reported regarding monocyte chemoattractant protein-1 (MCP-1) (34, 36) and IL-1 receptor antagonist (IL-1Ra) (17, 34), both showing a case of reduction and a case of no change.

### Risk of Bias and Quality of Evidence

In terms of the risk of bias in individual studies, four RCTs were considered as low risk, as most of the aspects were well-managed (16, 27, 35, 39). The randomization process and selection of the reported result were evaluated as the most common risks. The detailed outcomes for each bias domain are presented in Supplementary Table 4. There was no significant visual evidence of publication bias across the studies when the funnel plots were inspected (Supplementary Figure 2). Egger's regression and Begg's rank test also showed no statistical evidence of publication bias across studies on inflammatory markers (Supplementary Table 5). Quality of evidence on overall meta-analyzed markers is presented in Table 4, indicating moderate quality for CRP (downgraded by risk of bias), low quality for IL-6 and TNF-α (both downgraded by risk of bias and imprecision), and very low quality for IL-8 (downgraded by risk of bias, imprecision, and inconsistency).

## Discussion

The aim of the current study was to provide evidence of a correlation between oat consumption and inflammatory markers. According to this meta-analysis, there were no significant alterations in systemic inflammatory markers after oat consumption, although a meaningful proportion of systematically reviewed individual studies reported otherwise. However, when stratified based on the specific type of measurement method (hs-CRP), the SMD was negatively correlated with oat consumption. There was a significant decrease in CRP among subjects with one or more health complications. Similarly, IL-6 levels were significantly lower in subjects with dyslipidemia.

The physicochemical characteristics of β-glucan, such as molecular weight and structure, are related to immunomodulatory responses (46). Several laboratory experiments have investigated oat β-glucan to determine its correlation with inflammation. Kopiasz et al. (47) suggested that β-glucan potentially modulated the pathophysiology of inflammatory bowel disease in mouse models by altering the expression of pattern recognition receptors, including toll-like receptors and Dectin-1. Likewise, oat β-glucan intake inhibited a sudden surge of inflammatory markers like IL-10 and IL-12 in rats with lipopolysaccharide-induced enteritis (48). This was partially in line with our findings, i.e., unhealthy subjects, especially those with a high risk of inflammatory complications such as coronary heart disease (CHD) and type 2 diabetes mellitus, were more responsive to the effects of oats on systemic inflammatory markers. Multiple studies have also revealed the anti-inflammatory properties of avenanthramide (49–51), which allosterically suppresses the inhibitor of nuclear factor kappa B (IκB) kinase, leading to the prevention of IκB phosphorylation. This makes IκB resistant to degradation by the S26 proteasome, thereby inhibiting the nuclear factor kappa B (NF-κB) pathway. As reviewed in the current study, avenanthramide intake significantly reduced exercise-induced CRP and TNF-α levels in clinical settings (16, 27). Zhang et al. (34) further revealed that IL-1Ra, soluble vascular cell adhesion molecule-1 (sVCAM-1), G-CSF, and NRB levels significantly decreased following avenanthramide intake. Although our meta-analysis did not reveal a significant improvement in inflammatory markers, reduction considerably exceeded elevation in all reviewed RCTs. Notably, in addition to CRP and ILs, almost all inflammatory markers did not change significantly or were significantly reduced, as shown in Figure 2. Hence, further clinical trials exploring the effects of oat consumption on these markers can provide meaningful outcomes. Regarding IL-6, we observed that the change in the overall SMD was affected by the inclusion of a study by Nieman et al. (17), and the overall SMD reduction was statistically significant in the absence of this study. Notably, this was the only study with a 2-week long intervention period. Other studies had an intervention period that lasted for 3 or more weeks. This study employed a reversed outcome compared to previous meta-analyses on WG, where the longer intervention periods did not show a stronger tendency for IL-6 reduction (14, 19, 20). Considering that the study by Nieman et al. (17) also introduced serious heterogeneity, the reduction in IL-6 levels upon oat intake should not be negligible. In contrast to IL-6, our results corresponded with previous meta-analyses on WG regarding TNF-α levels, which remained unchanged (14, 19, 20).

Hs-CRP is an inflammatory marker used to evaluate and screen cardiac complications, including CHD (52). Oat intake was negatively correlated with hs-CRP serum levels according to the current meta-analysis, indicating that oat intake may attenuate the risk of cardiovascular disease (CVD). Multiple studies have reported a correlation between oat intake and reduced CVD risk. For example, oat β-glucan prevents both primary and secondary events of CHD (53, 54). In addition, a longitudinal study by Xu et al. showed a negative correlation between oat consumption in both heart disease and stroke, especially in the elderly (55). These effects are in accordance with the fact that the fiber-rich fraction of oats improves blood cholesterol levels (56, 57). Mechanistically, avenanthramide, a component of oats, prevents coronary plaque formation by inhibiting NF-κB in human aortic endothelial cells (HAECs) (51). Moreover, the secretion of IL-1β-induced pro-inflammatory cytokines, such as ICAM-1, VCAM-1, and E-selectin, was significantly reduced when HAECs were pre-incubated with 20 and 40 ng/mL of avenanthramide (58). The Food and Drug Administration of the United States has approved a health claim that consumption of the soluble fiber form of oat may attenuate the risk for CHD. Major evidence for this claim was based on the cumulative information on improvement in cholesterol levels (59). The current meta-analysis is in line with this health claim, as many of the reference RCTs specifically emphasize that their intervention in the oat group contains a high proportion of fiber. Moreover, our data provide more powerful evidence of the prophylactic effect of oat on CHD since hs-CRP is more highly associated with CHD than with other markers, including LDL (60).

The results of the meta-analysis on CRP and IL-6 suggested that unhealthy subjects were more responsive to the effects of oat consumption. Notably, a similar pattern was found in a previous meta-analysis on WG intake, which showed that the subgroup of unhealthy individuals showed a significant reduction in CRP and IL-6 levels after observing insignificant changes in the overall CRP and IL-6 levels (20). This indicates that oat intake does not simply reduce inflammatory markers. However, it modulates inflammatory marker level to be within the optimal range. In a Sprague Dawley rat model, oat β-glucan intake reversed the lipopolysaccharide (LPS)-induced upregulation of inflammatory markers, including IL-10 and IL-12 (48). However, there was no significant alteration in rats in a physiologically healthy state, except in the case where high-molecular-weight oat β-glucan increased IL-10 levels. Similarly, oat β-glucan intake partially reversed TNF-α, IL-6, and IL-1β levels in the liver tissue of an LPS-induced nonalcoholic steatohepatitis mouse model (61). *Ex vivo* experiments on a human endotoxemia model revealed that β-glucan enhanced the cytokine-producing attributes of LPS-induced tolerant monocytes, restoring the innate immune response (62). Accordingly, consumption of oats, especially the fiber-rich fraction, controls the immune response and helps sustain cytokine homeostasis.

There are several limitations worth noting in the current systematic review and meta-analysis. First, we were unable to investigate the dose-dependent correlation between the amount of oat intake and inflammatory marker levels due to limited access to the original data. This was mainly because the included studies did not specify the whole or dried weight of oats. Second, the imputed data (ρ = 0.5) were at risk of providing a distorted outcome. Although sensitivity analysis with ρ = 0.2 and 0.8 confirmed the robustness of the outcome, this issue should have been addressed when interpreting the data. The small number of meta-analyzed RCTs from those systematically reviewed overall was another limitation of our study. We also note that there may be a potential language bias, as only databases containing English literature were screened for reference collection. Finally, caution is needed regarding the heterogeneity in blood levels of outcome variables and other factors within the study design, such as population characteristics, control intervention, and type of blood sample (plasma vs. serum).

In summary, based on our meta-analysis results, there is insufficient evidence that oat intake reduces inflammatory response. However, a reduction in the levels of CRP, ILs, TNF-α, and other systemic markers was observed after oat intake according to the qualitatively synthesized data. A significant reduction in hs-CRP in the subgroup analysis can be potentially explained by the fact that oat intake is negatively correlated with CVD. Moreover, CRP and IL-6 levels decline in unhealthy subjects. Nevertheless, these findings can be confirmed by more robust studies in the future by including a large sample size. Therefore, future studies with properly presented outcomes are required to reach a stronger conclusion.

## Data Availability Statement

The original contributions presented in the study are included in the article/Supplementary Material, further inquiries can be directed to the corresponding author/s.

## Author Contributions

SunK, CJ, SungK, and SL: conceptualization. SunK, CJ, NA, and SukK: data screening and collection. SunK, CJ, and SP: data analysis. SunK and CJ: quality assessment. SunK, CJ, and SL: manuscript writing. All authors confirmed the manuscript and agreed to the submitted version.

## Conflict of Interest

The authors declare that the research was conducted in the absence of any commercial or financial relationships that could be construed as a potential conflict of interest.

## Publisher's Note

All claims expressed in this article are solely those of the authors and do not necessarily represent those of their affiliated organizations, or those of the publisher, the editors and the reviewers. Any product that may be evaluated in this article, or claim that may be made by its manufacturer, is not guaranteed or endorsed by the publisher.
