# Supplementary material for: Effects of Oats (Avena sativa L.) on Inflammation: A Systematic Review and Meta-Analysis of Randomized Controlled Trials
Source: Front Nutr. 2021 Aug 27;8:722866. doi: 10.3389/fnut.2021.722866 (PMC8429797; doi:10.3389/fnut.2021.722866)
Supplement: Supplementary file 1 [file Data_Sheet_1.docx]

Supplementary Material

## Supplementary Figures
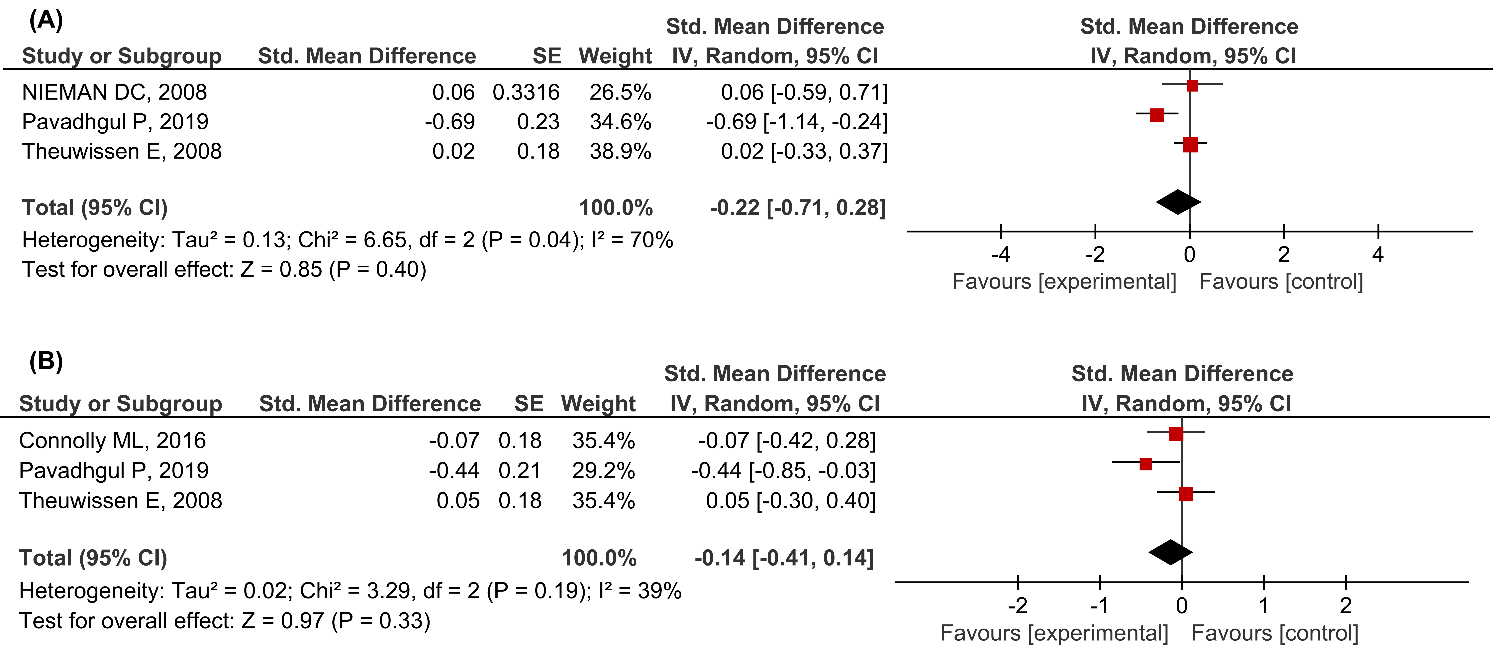


**Supplementary Figure 1.** Meta-analysis results of (A) IL-8 and (B) TNF-α.


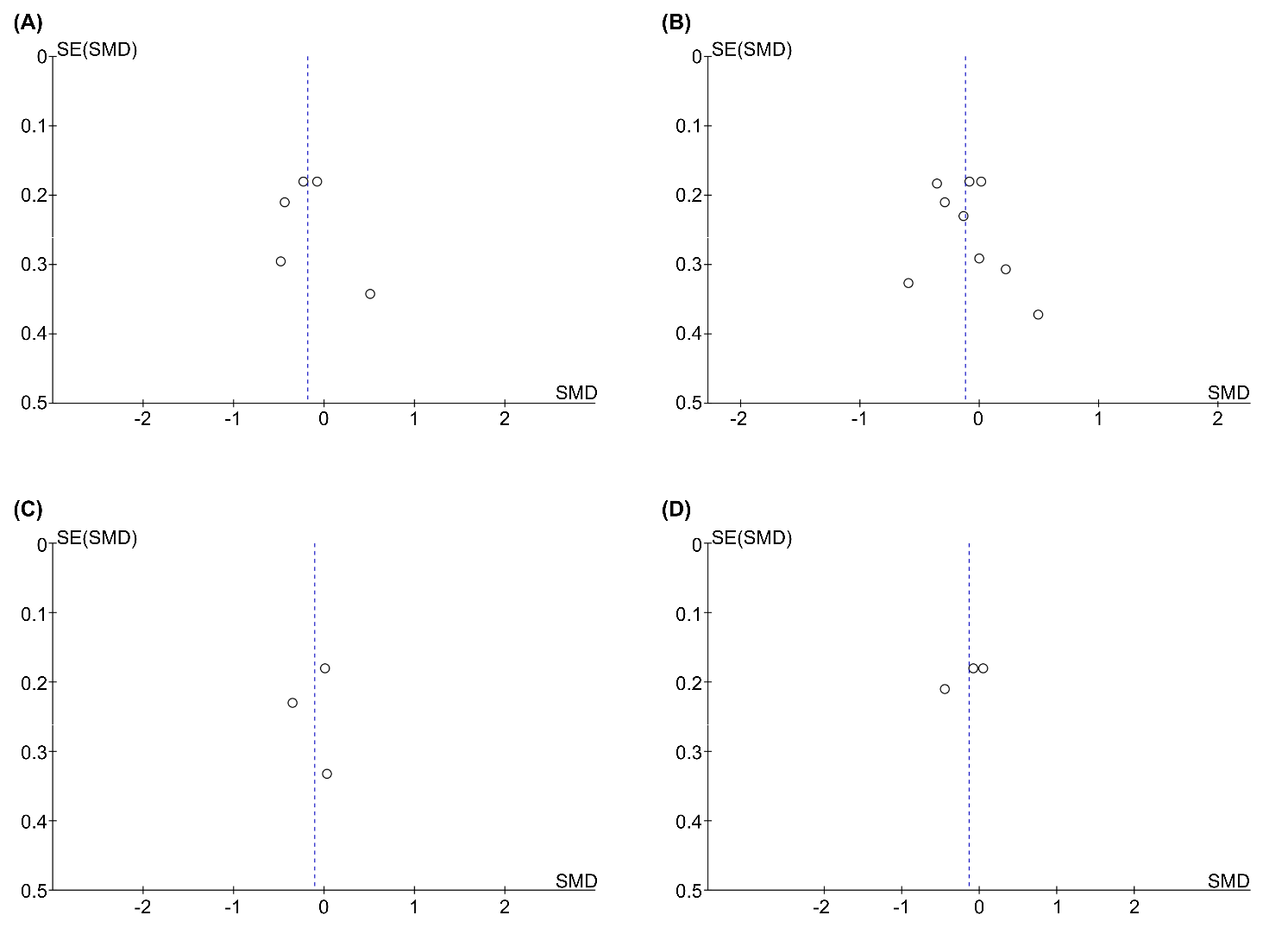


**Supplementary Figure 2.** The results of the funnel plot analysis: (A) IL-6, (B) CRP, (C) TNF-α, and (D) IL-8.

## Supplementary Tables

| **Section and Topic** | **Item #** | **Checklist item** | **Location where item is reported** |
| --- | --- | --- | --- |
| **TITLE** | | | |
| Title | 1 | Identify the report as a systematic review. | Page 1 |
| **ABSTRACT** | | | |
| Abstract | 2 | See the PRISMA 2020 for Abstracts checklist. | Page 1 |
| **INTRODUCTION** | | | |
| Rationale | 3 | Describe the rationale for the review in the context of existing knowledge. | Page 2 |
| Objectives | 4 | Provide an explicit statement of the objective(s) or question(s) the review addresses. | Page 2 |
| **METHODS** | | | |
| Eligibility criteria | 5 | Specify the inclusion and exclusion criteria for the review and how studies were grouped for the syntheses. | Pages 2-3 |
| Information sources | 6 | Specify all databases, registers, websites, organisations, reference lists and other sources searched or consulted to identify studies. Specify the date when each source was last searched or consulted. | Page 2 |
| Search strategy | 7 | Present the full search strategies for all databases, registers and websites, including any filters and limits used. | Page 2, Table 1 |
| Selection process | 8 | Specify the methods used to decide whether a study met the inclusion criteria of the review, including how many reviewers screened each record and each report retrieved, whether they worked independently, and if applicable, details of automation tools used in the process. | Pages 2-3 |
| Data collection process | 9 | Specify the methods used to collect data from reports, including how many reviewers collected data from each report, whether they worked independently, any processes for obtaining or confirming data from study investigators, and if applicable, details of automation tools used in the process. | Page 2 |
| Data items | 10a | List and define all outcomes for which data were sought. Specify whether all results that were compatible with each outcome domain in each study were sought (e.g. for all measures, time points, analyses), and if not, the methods used to decide which results to collect. | Pages 2-3 |
|  | 10b | List and define all other variables for which data were sought (e.g. participant and intervention characteristics, funding sources). Describe any assumptions made about any missing or unclear information. | Pages 2-3 |
| Study risk of bias assessment | 11 | Specify the methods used to assess risk of bias in the included studies, including details of the tool(s) used, how many reviewers assessed each study and whether they worked independently, and if applicable, details of automation tools used in the process. | Page 4 |
| Effect measures | 12 | Specify for each outcome the effect measure(s) (e.g. risk ratio, mean difference) used in the synthesis or presentation of results. | Pages 3-4 |
| Synthesis methods | 13a | Describe the processes used to decide which studies were eligible for each synthesis (e.g. tabulating the study intervention characteristics and comparing against the planned groups for each synthesis (item #5)). | Page 3 |
|  | 13b | Describe any methods required to prepare the data for presentation or synthesis, such as handling of missing summary statistics, or data conversions. | Pages 3-4, Table 2 |
|  | 13c | Describe any methods used to tabulate or visually display results of individual studies and syntheses. | Pages 3-4 |
|  | 13d | Describe any methods used to synthesize results and provide a rationale for the choice(s). If meta-analysis was performed, describe the model(s), method(s) to identify the presence and extent of statistical heterogeneity, and software package(s) used. | Pages 3-4 |
|  | 13e | Describe any methods used to explore possible causes of heterogeneity among study results (e.g. subgroup analysis, meta-regression). | Pages 3-4 |
|  | 13f | Describe any sensitivity analyses conducted to assess robustness of the synthesized results. | Page 3 |
| Reporting bias assessment | 14 | Describe any methods used to assess risk of bias due to missing results in a synthesis (arising from reporting biases). | Page 4 |
| Certainty assessment | 15 | Describe any methods used to assess certainty (or confidence) in the body of evidence for an outcome. | Page 4 |
| **RESULTS** | | | |
| Study selection | 16a | Describe the results of the search and selection process, from the number of records identified in the search to the number of studies included in the review, ideally using a flow diagram. | Page 4, Figure 1 |
|  | 16b | Cite studies that might appear to meet the inclusion criteria, but which were excluded, and explain why they were excluded. | Page 4, Figure 1 |
| Study characteristics | 17 | Cite each included study and present its characteristics. | Page 4 |
| Risk of bias in studies | 18 | Present assessments of risk of bias for each included study. | Page 6, Table 3, Supplementary Table 4 |
| Results of individual studies | 19 | For all outcomes, present, for each study: (a) summary statistics for each group (where appropriate) and (b) an effect estimate and its precision (e.g. confidence/credible interval), ideally using structured tables or plots. | Pages 4-5, Table 3 |
| Results of syntheses | 20a | For each synthesis, briefly summarise the characteristics and risk of bias among contributing studies. | Pages 4-5, Table 3 |
|  | 20b | Present results of all statistical syntheses conducted. If meta-analysis was done, present for each the summary estimate and its precision (e.g. confidence/credible interval) and measures of statistical heterogeneity. If comparing groups, describe the direction of the effect. | Pages 4-5, Tables 3 and 4 |
|  | 20c | Present results of all investigations of possible causes of heterogeneity among study results. | Pages 4-5, Table 4 |
|  | 20d | Present results of all sensitivity analyses conducted to assess the robustness of the synthesized results. | Pages 4-5, Supplementary Tables 2 and 3 |
| Reporting biases | 21 | Present assessments of risk of bias due to missing results (arising from reporting biases) for each synthesis assessed. | Page 6, Supplementary Figure 2, Supplementary Table 5 |
| Certainty of evidence | 22 | Present assessments of certainty (or confidence) in the body of evidence for each outcome assessed. | Page 6, Table 4 |
| **DISCUSSION** | | | |
| Discussion | 23a | Provide a general interpretation of the results in the context of other evidence. | Pages 6-7 |
|  | 23b | Discuss any limitations of the evidence included in the review. | Pages 7-8 |
|  | 23c | Discuss any limitations of the review processes used. | Pages 7-8 |
|  | 23d | Discuss implications of the results for practice, policy, and future research. | Page 8 |
| **OTHER INFORMATION** | | | |
| Registration and protocol | 24a | Provide registration information for the review, including register name and registration number, or state that the review was not registered. | Page 2 |
|  | 24b | Indicate where the review protocol can be accessed, or state that a protocol was not prepared. | Page 2 |
|  | 24c | Describe and explain any amendments to information provided at registration or in the protocol. | NA |
| Support | 25 | Describe sources of financial or non-financial support for the review, and the role of the funders or sponsors in the review. | Page 8 |
| Competing interests | 26 | Declare any competing interests of review authors. | Page 8 |
| Availability of data, code and other materials | 27 | Report which of the following are publicly available and where they can be found: template data collection forms; data extracted from included studies; data used for all analyses; analytic code; any other materials used in the review. | NA |

**Supplementary Table 1.** PRISMA checklist 2020.

| **Study group** | **Studies** | **Effect Estimate** | | **Heterogeneity** | | | **p-between group** |
| --- | --- | --- | --- | --- | --- | --- | --- |
|  |  | **Standard mean difference (95% CI)** | **p-value** | ***I*^2^ (%)** | **Q statistic** | **p-within group** |  |
| **CRP** | | | | | | | |
| Overall | 9 | -0.13 [-0.29, 0.04] | 0.14 | 0 | 6.95 | 0.54 |  |
| Type of measurement |  | | | | | | 0.14 |
| CRP | 2 | 0.14 [-0.25, 0.52] | 0.49 | 0 | 0.89 | 0.34 |  |
| hs-CRP | 7 | -0.19 [-0.37, -0.00] | **0.05** | 0 | 3.85 | 0.70 |  |
| Basal condition |  | | | | | | 0.21 |
| Healthy | 2 | 0.08 [-0.28, 0.44] | 0.67 | 0 | 0.17 | 0.68 |  |
| Unhealthy | 7 | -0.18 [-0.37, 0.01] | 0.06 | 0 | 5.23 | 0.52 |  |
| Type of oat product |  | | | | | | 0.87 |
| Whole | 4 | -0.13 [-0.42, 0.17] | 0.40 | 33 | 4.51 | 0.21 |  |
| Fiber-rich fraction | 5 | -0.09 [-0.33, 0.14] | 0.43 | 0 | 2.30 | 0.68 |  |
| Type of control |  | | | | | | 0.92 |
| Placebo/no intervention | 6 | -0.12 [-0.36, 0.11] | 0.31 | 18 | 6.09 | 0.30 |  |
| Other materials such as wheat | 3 | -0.10 [-0.37, 0.17] | 0.45 | 0 | 0.81 | 0.67 |  |
| **IL-6** | | | | | | | |
| Overall | 5 | -0.17 [-0.43, 0.08] | 0.18 | 18 | 4.87 | 0.30 |  |
| Basal condition |  | | | | | | 0.12 |
| Healthy | 2 | 0.11 [-0.37, 0.58] | 0.65 | 33 | 1.50 | 0.22 |  |
| Unhealthy | 3 | -0.34 [-0.63, -0.04] | **0.02** | 0 | 0.40 | 0.82 |  |
| Type of oat product |  | | | | | | 0.68 |
| Whole | 2 | -0.23 [-0.57, 0.12] | 0.19 | 0 | 0.97 | 0.32 |  |
| Fiber-rich fraction | 3 | -0.11 [-0.55, 0.33] | 0.63 | 47 | 3.75 | 0.15 |  |
| Type of control |  | | | | | | 0.62 |
| Placebo/no intervention | 2 | -0.01 [-0.82, 0.80] | 0.98 | 71 | 3.43 | 0.06 |  |
| Other materials such as wheat | 3 | -0.23 [-0.50, 0.04] | 0.10 | 0 | 0.97 | 0.61 |  |
| **IL-8** | 3 | -0.22 [-0.70, 0.27] | 0.37 | 49 | 3.95 | 0.14 |  |
| **TNF-α** | 3 | -0.12 [-0.40, 0.15] | 0.37 | 0 | 2.00 | 0.37 |  |

**Supplementary Table 2.** Meta-analysis summarization on CRP, IL-6, IL-8 and TNF-α (ρ = 0.2).

| **Study group** | **Studies** | **Effect Estimate** | | **Heterogeneity** | | | **p-between group** |
| --- | --- | --- | --- | --- | --- | --- | --- |
|  |  | **Standard mean difference (95% CI)** | **p-value** | ***I*^2^ (%)** | **Q statistic** | **p-within group** |  |
| **CRP** | | | | | | | |
| Overall | 9 | -0.12 [-0.30, 0.06] | 0.18 | 49 | 15.60 | 0.05 |  |
| Type of measurement |  | | | | | | 0.15 |
| CRP | 2 | 0.17 [-0.30, 0.65] | 0.47 | 47 | 1.90 | 0.17 |  |
| hs-CRP | 7 | -0.20 [-0.39, -0.01] | **0.04** | 38 | 9.74 | 0.14 |  |
| Basal condition |  | | | | | | 0.11 |
| Healthy | 2 | 0.05 [-0.16, 0.27] | 0.62 | 0 | 0.63 | 0.43 |  |
| Unhealthy | 7 | -0.19 [-0.40, 0.02] | 0.07 | 48 | 11.44 | 0.08 |  |
| Type of oat product |  | | | | | | 0.91 |
| Whole | 4 | -0.11 [-0.39, 0.16] | 0.42 | 62 | 7.94 | 0.05 |  |
| Fiber-rich fraction | 5 | -0.14 [-0.42, 0.15] | 0.36 | 48 | 7.62 | 0.11 |  |
| Type of control |  | | | | | | 0.97 |
| Placebo/no intervention | 6 | -0.12 [-0.46, 0.23] | 0.50 | 59 | 12.29 | 0.03 |  |
| Other materials such as wheat | 3 | -0.11 [-0.29, 0.06] | 0.21 | 37 | 3.15 | 0.21 |  |
| **IL-6** | | | | | | | |
| Overall | 5 | -0.20 [-0.48, 0.08] | 0.15 | 71 | 13.98 | 0.007 |  |
| Basal condition |  | | | | | | 0.12 |
| Healthy | 2 | 0.24 [-0.51, 1.00] | 0.53 | 78 | 4.64 | 0.03 |  |
| Unhealthy | 3 | -0.37 [-0.58, -0.16] | **0.0006** | 31 | 2.89 | 0.24 |  |
| Type of oat product |  | | | | | | 0.71 |
| Whole | 2 | -0.25 [-0.59, 0.09] | 0.15 | 74 | 3.91 | 0.05 |  |
| Fiber-rich fraction | 3 | -0.11 [-0.75, 0.52] | 0.72 | 80 | 9.98 | 0.007 |  |
| Type of control |  | | | | | | 0.76 |
| Placebo/no intervention | 2 | -0.02 [-1.41, 1.37] | 0.98 | 90 | 9.72 | 0.002 |  |
| Other materials such as wheat | 3 | -0.24 [-0.44, -0.05] | **0.02** | 49 | 3.92 | 0.14 |  |
| **IL-8** | 3 | -0.22 [-0.77, 0.33] | 0.43 | 87 | 15.99 | 0.0003 |  |
| **TNF-α** | 3 | -0.15 [-0.43, 0.13] | 0.30 | 76 | 8.17 | 0.02 |  |

**Supplementary Table 3.** Meta-analysis summarization on CRP, IL-6, IL-8 and TNF-α (ρ = 0.8).

| **Author** | **1** | **2** | **3** | **4** | **5** | **Overall** |
| --- | --- | --- | --- | --- | --- | --- |
| **McGeoch SC et al., 2013, United Kingdom (1)** | L | S | L | L | S | S |
| **Koenig RT et al., 2016, United States (2)** | S | L | L | L | L | L |
| **Koenig R et al., 2014, United States (3)** | S | L | L | L | L | L |
| **Zhang T et al., 2020, United States (4)** | S | H | H | L | S | H |
| **Nieman DC et al., 2008, United States (5)** | S | S | L | L | S | S |
| **Theuwissen E et al., 2009, Netherlands (6)** | S | H | L | L | S | H |
| **Tighe P et al., 2010, United Kingdom (7)** | L | S | L | L | S | S |
| **Fazilaty Z et al., 2018, Iran (8)** | S | S | L | L | S | S |
| **Ma X et al., 2013, China (9)** | S | L | L | L | S | S |
| **Ganda Mall JP et al., 2020, Sweden (10)** | L | S | L | L | S | S |
| **Xia Z et al., 2018, China (11)** | S | L | L | L | L | L |
| **Sirtori CR et al., 2012, Italy (12)** | S | H | L | L | S | H |
| **Connolly ML et al., 2016, United Kingdom (13)** | L | L | L | L | S | L |
| **Thompson JC et al., 2021, United States (14)** | S | S | S | H | S | H |
| **Pavadhgul P et al., 2019, Thailand (15)** | H | L | L | L | S | H |
| **Zhang X et al., 2014, United Kingdom (16)** | H | H | H | L | S | H |
| **Sawicki CM et al., 2016, United States (17)** | L | S | L | L | S | S |
| **Biörklund M et al., 2008, Sweden (18)** | S | L | L | L | S | S |
| **Sturtzel B et al., 2010, Austria (19)** | S | L | L | L | S | S |
| **Queenan KM et al., 2007, United States (20)** | S | S | S | L | S | S |
| **Cugnet-Anceau C et al., 2010, France (21)** | S | L | L | L | S | S |
| **Wolever TMS et al., 2010, Canada (22)** | L | S | S | L | S | S |
| **Maki KC et al., 2010, United States (23)** | S | S | S | L | S | S |
| 1: randomization process, 2: intended interventions (effect of assignment to intervention), 3: missing outcome data, 4: measurement of the outcome, 5: selection of the reported result, H: high risk, S: some concerns, L: low concerns | | | | | | |

**Supplementary Table 4.** Quality assessment outcome based on the second version of the Cochrane risk of bias tool for randomized trials (RoB2).

|  | **IL-6** | **CRP** | **TNF-α** | **IL-8** |
| --- | --- | --- | --- | --- |
| **Egger's test** | 0.61 | 0.44 | 0.17 | 0.93 |
| **Begg's test** | 0.81 | 0.47 | 0.30 | 0.60 |

**Supplementary Table 5.** *P* values of Egger's regression test and Begg's rank test for publication bias.

**References**

1. McGeoch SC, Johnstone AM, Lobley GE, Adamson J, Hickson K, Holtrop G, et al. A randomized crossover study to assess the effect of an oat-rich diet on glycaemic control, plasma lipids and postprandial glycaemia, inflammation and oxidative stress in Type 2 diabetes. *Diabet Med* (2013) 30(11):1314-23. Epub 2013/05/15. doi: 10.1111/dme.12228. PubMed PMID: 23668675; PubMed Central PMCID: PMCPMC4232050.

2. Koenig RT, Dickman JR, Kang CH, Zhang T, Chu YF, Ji LL. Avenanthramide supplementation attenuates eccentric exercise-inflicted blood inflammatory markers in women. *Eur J Appl Physiol* (2016) 116(1):67-76. Epub 2015/08/21. doi: 10.1007/s00421-015-3244-3. PubMed PMID: 26289619.

3. Koenig R, Dickman JR, Kang C, Zhang T, Chu Y-F, Ji LL. Avenanthramide supplementation attenuates exercise-induced inflammation in postmenopausal women. *Nutrition Journal* (2014) 13(1):21. doi: 10.1186/1475-2891-13-21.

4. Zhang T, Zhao T, Zhang Y, Liu T, Gagnon G, Ebrahim J, et al. Avenanthramide supplementation reduces eccentric exercise-induced inflammation in young men and women. *Journal of the International Society of Sports Nutrition* (2020) 17(1):41. doi: 10.1186/s12970-020-00368-3.

5. Nieman DC, Henson DA, McMahon M, Wrieden JL, Davis JM, Murphy EA, et al. β-Glucan, Immune Function, and Upper Respiratory Tract Infections in Athletes. *Medicine & Science in Sports & Exercise* (2008) 40(8).

6. Theuwissen E, Plat J, Mensink RP. Consumption of oat β-glucan with or without plant stanols did not influence inflammatory markers in hypercholesterolemic subjects. *Molecular Nutrition & Food Research* (2009) 53(3):370-6. doi: https://doi.org/10.1002/mnfr.200800132.

7. Tighe P, Duthie G, Vaughan N, Brittenden J, Simpson WG, Duthie S, et al. Effect of increased consumption of whole-grain foods on blood pressure and other cardiovascular risk markers in healthy middle-aged persons: a randomized controlled trial. *Am J Clin Nutr* (2010) 92(4):733-40. Epub 2010/08/06. doi: 10.3945/ajcn.2010.29417. PubMed PMID: 20685951.

8. Fazilaty Z, Chenari H, Shariatpanahi ZV. Effect of ß-glucan on serum levels of IL-12, hs-CRP, and clinical outcomes in multiple-trauma patients: a prospective randomized study. *Ulus Travma Acil Cerrahi Derg* (2018) 24(4):287-93. Epub 2018/07/22. doi: 10.5505/tjtes.2017.34514. PubMed PMID: 30028484.

9. Ma X, Gu J, Zhang Z, Jing L, Xu M, Dai X, et al. Effects of Avena nuda L. on metabolic control and cardiovascular disease risk among Chinese patients with diabetes and meeting metabolic syndrome criteria: secondary analysis of a randomized clinical trial. *Eur J Clin Nutr* (2013) 67(12):1291-7. Epub 2013/10/17. doi: 10.1038/ejcn.2013.201. PubMed PMID: 24129363.

10. Ganda Mall JP, Fart F, Sabet JA, Lindqvist CM, Nestestog R, Hegge FT, et al. Effects of Dietary Fibres on Acute Indomethacin-Induced Intestinal Hyperpermeability in the Elderly: A Randomised Placebo Controlled Parallel Clinical Trial. *Nutrients* (2020) 12(7). Epub 2020/07/08. doi: 10.3390/nu12071954. PubMed PMID: 32629992; PubMed Central PMCID: PMCPMC7400264.

11. Xia Z, Cholewa JM, Dardevet D, Huang T, Zhao Y, Shang H, et al. Effects of oat protein supplementation on skeletal muscle damage, inflammation and performance recovery following downhill running in untrained collegiate men. *Food Funct* (2018) 9(9):4720-9. Epub 2018/08/11. doi: 10.1039/c8fo00786a. PubMed PMID: 30094437.

12. Sirtori CR, Triolo M, Bosisio R, Bondioli A, Calabresi L, De Vergori V, et al. Hypocholesterolaemic effects of lupin protein and pea protein/fibre combinations in moderately hypercholesterolaemic individuals. *Br J Nutr* (2012) 107(8):1176-83. Epub 2011/10/29. doi: 10.1017/s0007114511004120. PubMed PMID: 22032303.

13. Connolly ML, Tzounis X, Tuohy KM, Lovegrove JA. Hypocholesterolemic and Prebiotic Effects of a Whole-Grain Oat-Based Granola Breakfast Cereal in a Cardio-Metabolic "At Risk" Population. *Front Microbiol* (2016) 7:1675. Epub 2016/11/23. doi: 10.3389/fmicb.2016.01675. PubMed PMID: 27872611; PubMed Central PMCID: PMCPMC5098205.

14. Thompson JC, Kirby T, DiSilvestro RA. Oat beta glucans anti-inflammatory actions in leg exercise stress. *Nutrition & Food Science* (2021) 51(1):1-9. doi: 10.1108/NFS-01-2020-0011.

15. Pavadhgul P, Bumrungpert A, Harjani Y, Kurilich A. Oat porridge consumption alleviates markers of inflammation and oxidative stress in hypercholesterolemic adults. *Asia Pac J Clin Nutr* (2019) 28(2):260-5. Epub 2019/06/14. doi: 10.6133/apjcn.201906_28(2).0008. PubMed PMID: 31192555.

16. Zhang X, McGeoch SC, Megson IL, MacRury SM, Johnstone AM, Abraham P, et al. Oat-enriched diet reduces inflammatory status assessed by circulating cell-derived microparticle concentrations in type 2 diabetes. *Mol Nutr Food Res* (2014) 58(6):1322-32. Epub 2014/03/08. doi: 10.1002/mnfr.201300820. PubMed PMID: 24604886.

17. Sawicki CM, McKay DL, McKeown NM, Dallal G, Chen CYO, Blumberg JB. Phytochemical Pharmacokinetics and Bioactivity of Oat and Barley Flour: A Randomized Crossover Trial. *Nutrients* (2016) 8(12):813. doi: 10.3390/nu8120813. PubMed PMID: 27983687.

18. Biörklund M, Holm J, Onning G. Serum lipids and postprandial glucose and insulin levels in hyperlipidemic subjects after consumption of an oat beta-glucan-containing ready meal. *Ann Nutr Metab* (2008) 52(2):83-90. Epub 2008/03/13. doi: 10.1159/000121281. PubMed PMID: 18334815.

19. Sturtzel B, Dietrich A, Wagner KH, Gisinger C, Elmadfa I. The status of vitamins B6, B12, folate, and of homocysteine in geriatric home residents receiving laxatives or dietary fiber. *J Nutr Health Aging* (2010) 14(3):219-23. Epub 2010/03/02. doi: 10.1007/s12603-010-0053-6. PubMed PMID: 20191257.

20. Queenan KM, Stewart ML, Smith KN, Thomas W, Fulcher RG, Slavin JL. Concentrated oat beta-glucan, a fermentable fiber, lowers serum cholesterol in hypercholesterolemic adults in a randomized controlled trial. *Nutr J* (2007) 6:6. Epub 2007/03/28. doi: 10.1186/1475-2891-6-6. PubMed PMID: 17386092; PubMed Central PMCID: PMCPMC1847683.

21. Cugnet-Anceau C, Nazare JA, Biorklund M, Le Coquil E, Sassolas A, Sothier M, et al. A controlled study of consumption of beta-glucan-enriched soups for 2 months by type 2 diabetic free-living subjects. *Br J Nutr* (2010) 103(3):422-8. Epub 2009/09/29. doi: 10.1017/s0007114509991875. PubMed PMID: 19781120.

22. Wolever TM, Tosh SM, Gibbs AL, Brand-Miller J, Duncan AM, Hart V, et al. Physicochemical properties of oat β-glucan influence its ability to reduce serum LDL cholesterol in humans: a randomized clinical trial. *Am J Clin Nutr* (2010) 92(4):723-32. Epub 2010/07/28. doi: 10.3945/ajcn.2010.29174. PubMed PMID: 20660224.

23. Maki KC, Beiseigel JM, Jonnalagadda SS, Gugger CK, Reeves MS, Farmer MV, et al. Whole-grain ready-to-eat oat cereal, as part of a dietary program for weight loss, reduces low-density lipoprotein cholesterol in adults with overweight and obesity more than a dietary program including low-fiber control foods. *J Am Diet Assoc* (2010) 110(2):205-14. Epub 2010/01/28. doi: 10.1016/j.jada.2009.10.037. PubMed PMID: 20102847.
